# Comparative performance of GPT-4, GPT-o3, GPT-5, Gemini-3-Flash, and DeepSeek-R1 in ophthalmology question answering

**DOI:** 10.3389/fcell.2026.1744389

**Published:** 2026-01-29

**Authors:** Ping Zhang, Jiaoman Wang, Xinya Hu, Xiaoqing Wang, Xianming Fan, Wei Chi, Weihua Yang

**Affiliations:** 1 Department of Ophthalmology, Shenzhen People’s Hospital, The Second Clinical Medical College of Jinan University, Shenzhen, Guangdong, China; 2 Eye Hospital and School of Ophthalmology and Optometry, Wenzhou Medical University, Wenzhou, Zhejiang, China; 3 Shenzhen Eye Hospital, Shenzhen Eye Medical Center, Southern Medical University, Shenzhen, Guangdong, China

**Keywords:** artificial intelligence (AI), clinical decision support, large language model (LLM), medical education, ophthalmology

## Abstract

**Background:**

The application of large language models (LLMs) in medicine is rapidly advancing, showing particular promise in specialized fields like ophthalmology. However, existing research has predominantly focused on validating individual models, with a notable scarcity of systematic comparisons between multiple state-of-the-art LLMs.

**Objective:**

To systematically evaluate the performance of GPT-4, GPT-o3, GPT-5, Gemini-3-Flash, and DeepSeek-R1 on ophthalmology question-answering tasks, with a specific focus on response consistency and factual accuracy.

**Methods:**

A total of 300 single-best-answer multiple-choice questions were sampled from the StatPearls ophthalmology question bank. The questions were categorized into four difficulty levels (Levels 1–4) based on the inherent difficulty ratings provided by the database. Each model provided independent answers three times under two distinct prompting strategies: a direct neutral prompt and a role-based prompt. Fleiss’ kappa (κ) was used to assess inter-run response consistency, and overall accuracy was employed as the primary performance metric.

**Results:**

Accuracy: Gemini-3-Flash achieved the highest overall accuracy (83.3%), followed by GPT-o3 (79.2%) and DeepSeek-R1 (74.4%). GPT-4 (69.9%) and GPT-5 (69.1%) demonstrated the lowest accuracies. Consistency: GPT-o3 demonstrated the highest decision stability (κ = 0.966), followed by DeepSeek-R1 (κ = 0.904) and Gemini-3-Flash (κ = 0.860). GPT-5 exhibited the lowest stability (κ = 0.668). Influencing Factors: Prompting strategies did not significantly affect model accuracy. While Gemini-3-Flash remained stable across difficulty levels, DeepSeek-R1 and GPT-o3 showed enhanced relative performance on more complex tasks.

**Conclusion:**

GPT-o3 and Gemini-3-Flash achieve superior stability and accuracy in ophthalmology Question Answering (QA), making them suitable for high-stakes clinical decision support. The open-source model DeepSeek-R1 shows competitive potential, especially in complex tasks. Notably, GPT-5 failed to surpass its predecessor in both accuracy and consistency in this specialized domain. Prompt engineering has a limited impact on performance for closed-ended medical questions. Future work should extend to multimodal integration and real-world clinical validation to enhance the practical utility and reliability of LLMs in medicine.

## 1 Introduction

The application of artificial intelligence (AI) in medicine is being profoundly transformed by advances in large language models (LLMs) (Thirunavukarasu et al., 2023; Gilson et al., 2023; Ting et al., 2024; Dave et al., 2023). LLMs are generative AI systems trained on vast textual datasets comprising billions of parameters. Leveraging sophisticated conversational capabilities, these models demonstrate exceptional proficiency in analyzing complex linguistic structures and have shown significant efficacy in tasks requiring medical text comprehension and reasoning (Waisberg et al., 2023; Eriksen et al., 2024; Barile et al., 2024; Liu et al., 2023). Since the launch of ChatGPT in late 2022 (ChatGPT, 2025), research on the medical applications of LLMs has accelerated rapidly. Early models such as GPT-4 demonstrated performance on multiple knowledge assessment benchmarks that was comparable to medical experts. This progress provides a crucial foundation for deploying LLMs in highly specialized medical disciplines that demand precise knowledge expression, such as ophthalmology (Sakai et al., 2023; Tanaka et al., 2024; Lin et al., 2023; Jang et al., 2023; Takagi et al., 2023; Kung et al., 2023; Taloni et al., 2023; Li Z. et al., 2025).

In ophthalmology, LLMs have been explored for potential applications in disease diagnosis, clinical decision support, and medical education (Kurapati et al., 2025; Spina et al., 2025). Current research indicates that models like GPT-4 achieve accuracy comparable to that of senior ophthalmologists in question-answering tasks related to glaucoma, retinal diseases, uveitis, ocular surface disorders, and dry eye syndrome (Lyons et al., 2024; Huang et al., 2024; Zhao et al., 2024; Ling et al., 2025; Shi et al., 2024). The model also shows promising potential for application in subspecialty fields such as neuro-ophthalmology and corneal diseases (Barclay et al., 2024; Madadi et al., 2024). Although a limited number of studies have attempted to compare different LLMs (e.g., GPT-4o and DeepSeek) (Huang et al., 2025), revealing superior performance in accuracy and logical consistency for some, the existing literature has predominantly centered on validating the performance of single model families, particularly the GPT series (Waisberg et al., 2023; Demir, 2024; Moshirfar et al., 2023; Antaki et al., 2024; Milad et al., 2024). There remains a notable scarcity of systematic comparisons across the rapidly expanding landscape of state-of-the-art LLMs. This knowledge gap is becoming increasingly critical given the rapid emergence of new models with distinct architectures and reasoning capabilities. For instance, OpenAI’s GPT-o3 (released in April 2025) integrates implicit Chain-of-Thought (CoT) reasoning with large-scale reinforcement learning, substantially enhancing its performance on complex scientific problems (OpenAI, 2025). Concurrently, the advent of GPT-5 represents a significant generational leap in foundational capabilities, aiming to achieve superior generalization, reduced hallucination rates, and deeper semantic understanding compared to its predecessors (GPT, 2025). Furthermore, Google’s Gemini series has emerged as a formidable contender. Its native multimodal architecture and strong performance on reasoning benchmarks represent another distinct approach to medical knowledge processing (Team et al., 2023; Yang et al., 2024). In the open-source domain, models such as DeepSeek’s DeepSeek-R1 (Jan. 2025) employ a Mixture-of-Experts (MoE) architecture, reinforcement learning, and multi-stage training to achieve enhanced logical reasoning, a capability characterized by a transparent process that offers superior adaptability to evolving medical knowledge and enhanced interpretability for clinical decision-making (Deepseek, 2025).

Evaluation results suggest that DeepSeek-R1 outperforms mainstream large language models, including the GPT-4 series, on general benchmarks such as mathematical problem-solving and code generation (Gibney, 2025). This underscores fundamental differences in core cognitive abilities such as logical reasoning, multi-step problem-solving, and structured output generation. Given that ophthalmic diagnosis likewise relies heavily on rigorous reasoning and complex knowledge integration, these architectural and knowledge-update differences may theoretically lead to divergent performance in specialized medical settings. However, such hypotheses have not yet been substantiated by systematic validation in ophthalmology, largely due to limited access to cutting-edge proprietary models such as GPT-o3 and the very recent release of models like Gemini-3-Flash.

Building on these considerations, this study provides the first systematic evaluation of five cutting-edge models—GPT-4, GPT-5, GPT-o3, Gemini-3-Flash, and DeepSeek-R1—in ophthalmology-focused question answering. We employed the StatPearls (StatPearls, 2023) question bank, an authoritative resource widely used for resident examinations and clinical training, to construct a comprehensive evaluation framework. In contrast to prior studies that tested only a single model, this work offers the first comparative assessment of three frontier models in a specialized medical domain. Our results provide data on AI-assisted tool selection and inform the ongoing refinement of medical reasoning capabilities. Understanding these performance metrics offers insights into the application of these models within medical education and clinical decision-making.

## 2 Methods

Question Source: The questions used to evaluate the models were obtained from the Ace Ophthalmology exam on StatPearls (www.statpearls.com). This database comprises 3,004 ophthalmology-related questions, which are categorized into four levels of cognitive complexity: Level 1 (4 questions), Level 2 (340 questions), Level 3 (1,849 questions), and Level 4 (811 questions). Given the limited number of Level 1 questions (n = 4), this level was excluded from the analysis. Furthermore, all image-based questions from Levels 2, 3, and 4 were also excluded, as DeepSeek-R1 lacks the capability to interpret images without accompanying text. Following these exclusions, a stratified random sampling strategy was employed to select approximately 10% of the remaining text-based questions from each of Levels 2, 3, and 4 for assessment.

Prompting Technique: For each LLM and each question, two distinct prompting techniques were applied: a zero-shot forced-choice prompt (Prompt 0) and a role-based zero-shot plan-and-solve + prompt (Prompt 1). Each prompting technique was independently evaluated over three separate runs to assess the reproducibility and reliability of the outcomes. Furthermore, to mitigate potential learning biases resulting from contextual dependencies, each replication was conducted in a completely new chat session, thereby ensuring the independence of test results across replications. Prompt 0 was designed as a straightforward instruction: “Please read the question and select the single best answer from the options provided. Provide your reasoning.” In contrast, Prompt 1 was designed to simulate real-world clinical reasoning by assigning a specialized role to the model, thereby encouraging more detailed and contextualized analysis. It was formulated as follows: “You are an ophthalmology expert with advanced knowledge. I will present you with an ophthalmology question followed by several possible answers. Please read the question carefully, select one answer, and explain your reasoning. You must provide an answer and may not remain silent.”

Outcome measures: The primary objective of this study was to evaluate and compare the performance of five large language models—GPT-4, GPT-5, GPT-o3, Gemini-3-Flash, and DeepSeek-R1—on an ophthalmology knowledge question-answering task. To this end, two core evaluation dimensions were established: (1) response consistency, quantified by Fleiss’ kappa across three independent trials to assess decision stability and reliability (Landis and Koch, 1977); and (2) overall accuracy, calculated as the proportion of correct responses to evaluate the models’ factual proficiency. To further delineate performance, consistency and accuracy were analyzed across ten ophthalmology subspecialties. Additionally, stratified analyses were conducted to examine performance variations based on cognitive complexity (Levels 2, 3, and 4) and prompting strategies (direct neutral prompts [Prompt 0] *versus* role-based prompts [Prompt 1]).

Data Management: Data were managed using Microsoft Excel (Version 16.0; Microsoft Corp., Redmond, WA, United State) and analyzed using R (Version 4.3.2; R Foundation for Statistical Computing, Vienna, Austria) under the Windows 11 (64-bit) operating system. As the study did not involve human participants, neither ethical approval nor informed consent was required.

Statistical Analysis: For the 300 ophthalmic single-best-answer items, we calculated the overall accuracy (proportion correct) for each LLM. Differences in the number of correct responses across LLMs were compared using Pearson’s chi-square (χ^2^) test. If the assumptions of the chi-square test were violated (defined as more than 20% of cells having an expected count less than 5), Fisher’s exact test was employed instead to ensure robust inference. Similarly, Pearson’s chi-square tests (with Fisher’s exact test where appropriate) were used to compare model accuracies under the two prompt conditions, across the three difficulty levels (2, 3, and 4), and across the 10 distinct ophthalmic subspecialties. For significant omnibus tests involving more than two groups, post-hoc pairwise comparisons were conducted with a Bonferroni adjustment for multiple comparisons. A two-sided P-value of less than 0.05 was considered statistically significant. Additionally, Fleiss’ kappa was used to evaluate intra-model response consistency across the three independent runs for each LLM and each prompt, serving as a measure of decision-making stability.

## 3 Results

### 3.1 Assessment of intra-model response consistency

To assess the stability and reliability of each model’s outputs, we quantified within-model agreement using Fleiss’ kappa (κ) based on three independent responses per item from GPT-4, GPT-5, GPT-o3, DeepSeek-R1, and Gemini-3-Flash. Higher κ values indicate a greater tendency to reproduce the same answer given identical inputs, reflecting greater decision stability.

Our results show that all models demonstrated substantial agreement, with GPT-o3 achieving the highest overall consistency (κ = 0.966, 95% CI: 0.944–0.986), followed by DeepSeek-R1 (κ = 0.904, 95% CI: 0.869–0.933), Gemini-3-Flash (κ = 0.860, 95% CI: 0.810–0.901), and GPT-4 (κ = 0.842, 95% CI: 0.804–0.878). Notably, GPT-5 exhibited lower stability compared to its peers (κ = 0.668, 95% CI: 0.618–0.719). Analysis of task difficulty revealed a progressive decline in consistency as difficulty levels increased. Specifically, the kappa coefficient decreased from 0.885 (95% CI: 0.838–0.937) at Level 2 to 0.853 (95% CI: 0.830–0.875) at Level 3, and further to 0.801 (95% CI: 0.764–0.838) at Level 4. Despite this downward trend, all difficulty levels maintained a high degree of reliability, with Kappa values consistently exceeding the commonly accepted 0.80 threshold for almost perfect agreement.

Furthermore, the degree of agreement varied across different model–prompt combinations within each difficulty tier. At Level 2, most model–prompt configurations demonstrated high stability. GPT-o3 showed the highest reproducibility, achieving perfect agreement (κ = 1.00) with Prompt 0 and near-perfect agreement (κ = 0.95) with Prompt 1. DeepSeek-R1, GPT-4, and Gemini-3-Flash also maintained strong consistency (κ = 0.80–0.95). In contrast, GPT-5 exhibited the lowest stability within this tier, with kappa values ranging from 0.76 to 0.78 across prompts—below the “almost perfect” threshold reached by other models. At Level 3, the agreement scores exhibited a broad distribution. GPT-o3 maintained its lead in this tier, achieving the highest kappa coefficients (0.97–0.99). DeepSeek-R1 also demonstrated strong agreement, with values ranging from 0.91 to 0.95. In contrast, GPT-5 yielded the lowest scores (κ = 0.65–0.68), while the performance of the remaining models fell within an intermediate range (κ = 0.84–0.90).

**FIGURE 1:**
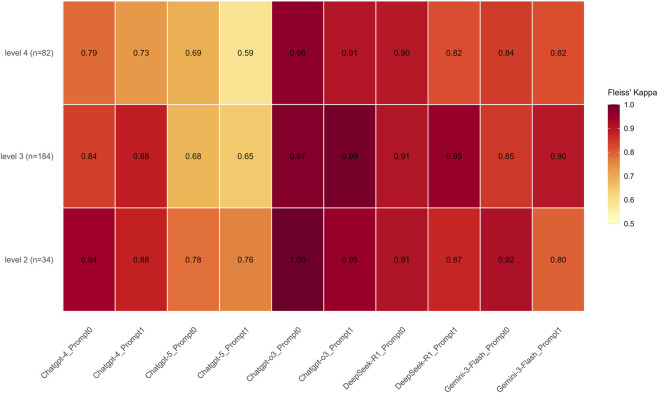
Fleiss’ kappa heatmap illustrating intra-model response consistency across models, difficulty levels, and prompting strategies. The heatmap displays Fleiss’ kappa (κ) values, representing agreement among three independent runs for each model–prompt combination across ophthalmology question difficulty levels (Levels 2–4). Higher κ values indicate greater within-model stability. GPT-o3 demonstrated the highest overall consistency, achieving perfect agreement (kappa = 1.00) at Level 2 and maintaining near-perfect scores (kappa = 0.91–0.99) across higher difficulty tiers. DeepSeek-R1 and Gemini-3-Flash followed with strong consistency, while GPT-4 showed a notable decline at Level 4 (kappa = 0.73–0.79). Notably, GPT-5 exhibited the lowest stability across all tiers, with consistency dropping to kappa = 0.59 at Level 4. In contrast, GPT-o3 maintained high reproducibility across all levels and prompts, suggesting more deterministic reasoning behavior. Color intensity corresponds to κ magnitude, with lighter and darker shades indicating lower and higher values, respectively.

At Level 4, the distribution of agreement scores widened further (κ = 0.59–0.96). GPT-o3 consistently demonstrated the highest reliability (κ = 0.91–0.96), followed by DeepSeek-R1 (κ = 0.82–0.90). Gemini-3-Flash recorded k values of 0.82 and 0.84, while GPT-4 yielded scores of 0.73 and 0.79. Consistent with observations at previous levels, GPT-5 exhibited the lowest stability, with k values declining to 0.59 and 0.69 (Figure 1).

### 3.2 Assessment of subspecialty-specific consistency

Across most subspecialties, GPT-o3 and DeepSeek-R1 demonstrated consistently high agreement, with kappa values generally ≥0.90 and frequent instances of perfect agreement (κ = 1.00) under both prompts. Gemini-3-Flash also exhibited strong overall consistency, achieving perfect agreement (κ = 1.00) in the Uveitis and Glaucoma subspecialties across both prompt configurations. Notably, in Ocular Pathology/Oncology, the agreement for Gemini-3-Flash improved from κ = 0.69 under Prompt 0 to κ = 1.00 under Prompt 1. Conversely, in Pediatric Ophthalmology/Strabismus, the kappa value decreased from 0.83 under Prompt 0 to 0.61 under Prompt 1.

**FIGURE 2:**
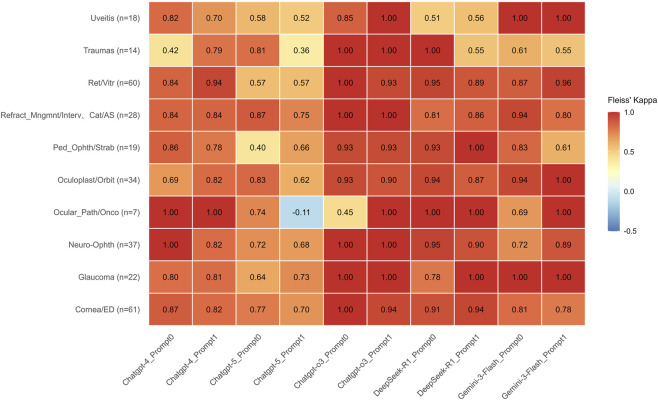
Comparison of Inter-rater Reliability Across Large Language Models in Ophthalmology Subspecialties. Heatmap of Fleiss’ Kappa (kappa) values across ten ophthalmology subspecialties under two prompting strategies. Overview of Agreement: The heatmap visualizes the level of agreement among LLM outputs. Values are color-coded according to the scale on the right. The heatmap illustrates that reasoning-optimized models, GPT-o3 and DeepSeek-R1, demonstrate superior and highly stable consistency, generally maintaining kappa ≥0.90 and frequently reaching perfect agreement (kappa = 1.00) regardless of the prompt used. Gemini-3-Flash also exhibits robust performance, particularly achieving kappa = 1.00 in Uveitis and Glaucoma, though it shows moderate sensitivity to prompting in Pediatric Ophthalmology/Strabismus (decreasing from 0.83 to 0.61). In stark contrast, GPT-5 displays significant performance volatility under Prompt 1, where agreement levels drop to moderate or low (0.36–0.57) across Uveitis, Traumas, and Retina/Vitreous subspecialties. Most notably, in Ocular Pathology/Oncology (n = 7), GPT-5 under Prompt 1 yields the only negative agreement value in the dataset (kappa = −0.11), whereas all other models achieved perfect agreement (kappa = 1.00) in this category under at least one prompt configuration. Sample sizes (n) for each subspecialty are provided in parentheses along the vertical axis.

In contrast, under Prompt 1, GPT-5 exhibited greater dispersion in agreement, with kappa values falling to moderate or low levels (0.36–0.57) across several subspecialties, including Uveitis, Trauma, and Retina/Vitreous. Notably, in the subspecialty of Ocular Pathology/Oncology (n = 7), GPT-5 recorded the lowest agreement (κ = −0.11) with Prompt 1. Under at least one prompt configuration, however, the other models achieved perfect agreement (κ = 1.00) in this same subspecialty (Figure 2).

### 3.3 Comparison of model accuracy

**FIGURE 3:**
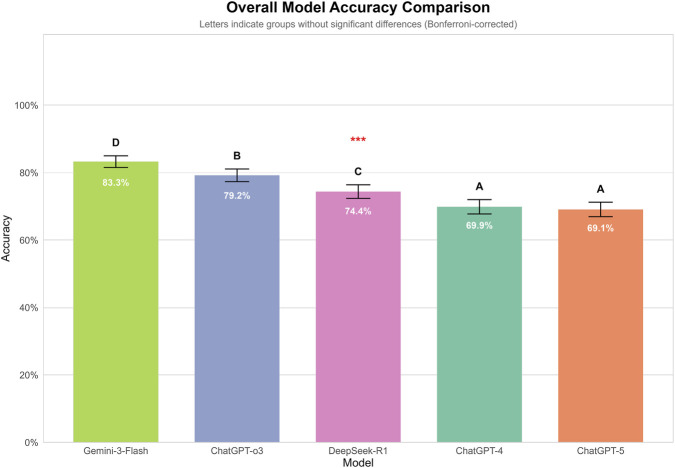
Overall accuracy of GPT-4, GPT-5, GPT-o3, Genimi-3-Flash, and DeepSeek-R1 on 300 ophthalmology multiple-choice questions. Each model answered questions independently under two prompting strategies across three independent runs. Overall, significant differences in accuracy were observed among the five models (p < 0.001). Gemini-3-Flash achieved the highest overall accuracy (83.3%), with significantly higher performance than all other models. This was followed by GPT-o3 (79.2%) and DeepSeek-R1 (74.4%). GPT-4 (69.9%) and GPT-5 (69.1%) demonstrated the lowest accuracies, with no statistically significant difference observed between them.

Each model independently answered 300 ophthalmology multiple-choice questions three times under two distinct prompting strategies, yielding a total of 9,000 responses. Overall, significant differences in accuracy were observed among the five models (p < 0.001). Gemini-3-Flash achieved the highest overall accuracy (83.3%), with significantly higher performance than all other models. This was followed by GPT-o3 (79.2%) and DeepSeek-R1 (74.4%). GPT-4 (69.9%) and GPT-5 (69.1%) demonstrated the lowest accuracies, with no statistically significant difference observed between them (Figure 3).

**FIGURE 4:**
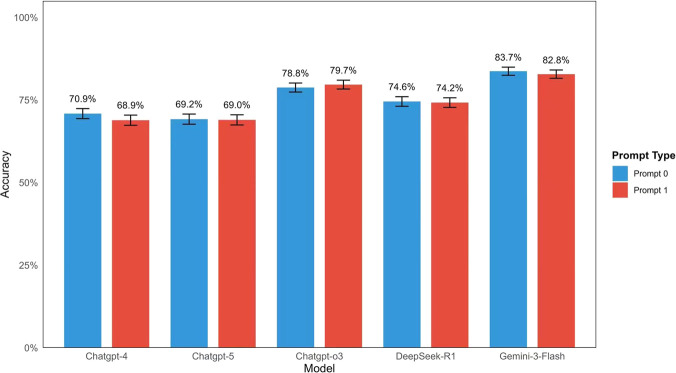
Comparison of Inter-rater Reliability Across Large Language Models in Ophthalmology Subspecialties. Heatmap of Fleiss' Kappa (k) values across ten ophthalmology subspecialties under two prompting strategies. For each LLM (GPT-4, GPT-5, GPT-o3, DeepSeek-R1, and Gemini-3-Flash) and each question, two distinct prompting techniques were applied: a zero-shot forced-choice prompt (Prompt 0) and a role-based zero-shot plan-and-solve + prompt (Prompt 1). Bars depict the number and percentage of correct answers out of 900 responses per model. No statistically significant differences in accuracy were observed between the two prompt types for any model: GPT-4 (70.9% vs. 68.9%, p = 0.382), GPT-5 (69.2% vs. 69.0%, p = 0.959), GPT-o3 (78.8% vs. 79.7%, p = 0.684), DeepSeek-R1 (74.6% vs. 74.2%, p = 0.914), and Gemini-3-Flash (83.7% vs. 82.8%, p = 0.658). These results indicate that prompt style had minimal impact on performance in this closed-ended task.

Further analysis of the effect of prompt strategies showed no statistically significant differences in accuracy between the simple prompt (Prompt 0) and the role-based prompt (Prompt 1) for any of the tested models. Specifically, the accuracy for GPT-4 was 70.9% with Prompt 0% and 68.9% with Prompt 1 (p = 0.382). For GPT-5, the results remained consistent across strategies, with 69.2% for Prompt 0% and 69.0% for Prompt 1 (p = 0.959). Similarly, for GPT-o3, accuracy was 78.8% with Prompt 0% and 79.7% with Prompt 1 (p = 0.684), while for DeepSeek-R1, it was 74.6% and 74.2%, respectively (p = 0.914). Finally, the highest-performing model, Gemini-3-Flash, achieved 83.7% with Prompt 0% and 82.8% with Prompt 1 (p = 0.658). These results are summarized in (Figure 4).

### 3.4 Model performance across difficulty levels

**FIGURE 5:**
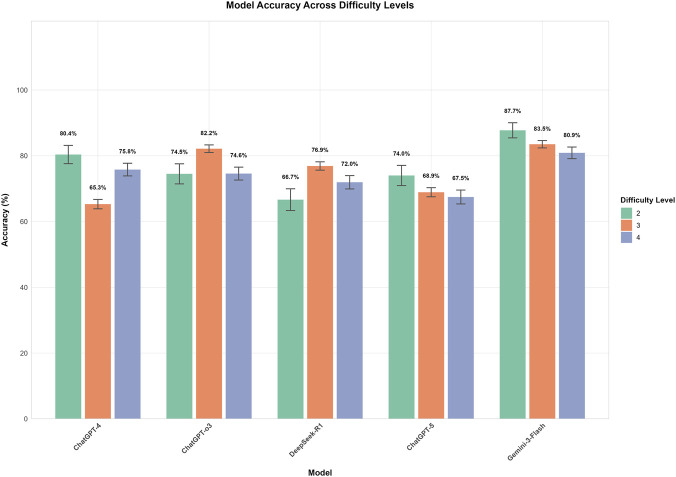
Model accuracy across different task difficulty Levels. This bar chart illustrates the mean accuracy (%) of five models (GPT-4, GPT-o3, DeepSeek-R1, GPT-5, and Gemini-3-Flash) across three difficulty levels (2, 3, and 4), with error bars representing Standard Error. Gemini-3-Flash exhibited the highest overall performance and maintained statistical stability across levels (p = 0.083), whereas GPT-4, GPT-o3, and DeepSeek-R1 showed significant performance fluctuations (all p < 0.01). Notably, while Gemini-3-Flash and GPT-4 peaked at Level 2, GPT-o3 and DeepSeek-R1 achieved their highest accuracy at Level 3; no significant decline was observed for GPT-5 despite a numerical downward trend (p = 0.231).

**FIGURE 6:**
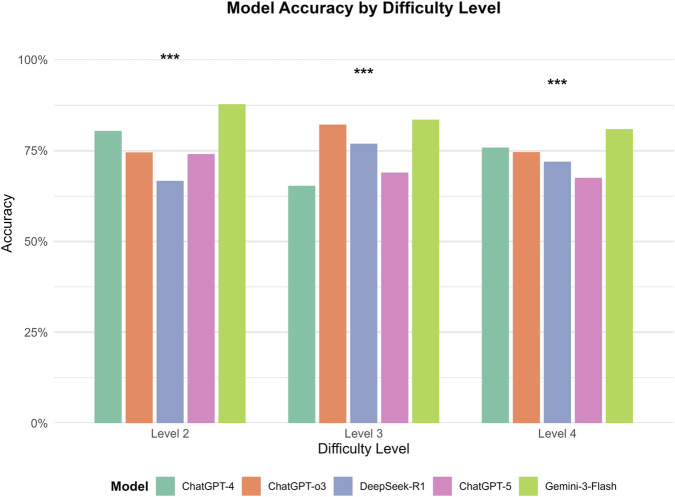
Between-model comparison of performance across difficulty Levels. This grouped bar chart illustrates the comparative accuracy (%) of five models (GPT-4, GPT-o3, DeepSeek-R1, GPT-5, and Gemini-3-Flash) segmented by difficulty levels 2, 3, and 4. Significant differences between models within each level were identified (***p < 0.001). Gemini-3-Flash consistently demonstrated superior performance, significantly outperforming GPT-o3, GPT-5, and DeepSeek-R1 at Level 2, and maintaining a significant lead over most models at Level 4, although its advantage over GPT-4 did not reach statistical significance at Level 2 (p = 0.058) or Level 4 (p = 0.063). At Level 3, both Gemini-3-Flash and GPT-o3 achieved comparable top-tier performance (p = 0.430), while significantly surpassing the remaining three models (p < 0.001).

Significant differences in performance were observed among the five models across difficulty levels. Within each model, accuracy varied significantly with difficulty for GPT-4, GPT-o3, and DeepSeek-R1 (all p < 0.01). However, no statistically significant differences were found across difficulty levels for GPT-5 (p = 0.231) or Gemini-3-Flash (p = 0.083), suggesting that their performance remained relatively stable across these levels. Gemini-3-Flash demonstrated the highest overall performance, peaking at Level 2 (87.7%) and maintaining high accuracy at Levels 3 (83.5%) and 4 (80.9%); post-hoc tests confirmed no significant differences between these levels. GPT-o3 performed best at Level 3 (82.2%), which was significantly higher than its performance at Level 2 (74.5%, p < 0.05) and Level 4 (74.6%, p < 0.01). In contrast, GPT-4 achieved its highest accuracy at Level 2 (80.4%), which decreased significantly to 65.3% at Level 3 (p < 0.001), before rebounding to 75.8% at Level 4 (p < 0.001). DeepSeek-R1 showed its lowest accuracy at Level 2 (66.7%), which increased significantly to 76.9% at Level 3 (p < 0.01). Finally, GPT-5 displayed a non-significant numerical decline as difficulty increased, from 74.0% at Level 2%–68.9% and 67.5% at Levels 3 and 4, respectively (Figure 5). Between-model comparisons within each difficulty level revealed statistically significant differences across three levels. Specifically, at Level 2, Gemini-3-Flash demonstrated the highest accuracy (87.7%). While its lead over GPT-4 (80.4%) was not significant (P = 0.058), Gemini-3-Flash significantly outperformed GPT-o3 (74.5%, P = 0.001), GPT-5 (74.0%, P < 0.001), and DeepSeek-R1 (66.7%, P < 0.001). At Level 3, Gemini-3-Flash (83.5%) and GPT-o3 (82.2%) achieved comparable top-tier performance (P = 0.430). Notably, both models significantly surpassed DeepSeek-R1 (76.9%, P < 0.001), GPT-5 (68.9%, P < 0.001), and GPT-4 (65.3%, P < 0.001). At Level 4, Gemini-3-Flash consistently maintained the leading position (80.9% accuracy), which was significantly higher than the performance of GPT-o3 (74.6%, P < 0.05), DeepSeek-R1 (72.0%, P < 0.01), and GPT-5 (67.5%, P < 0.001). However, the difference between Gemini-3-Flash and GPT-4 (75.8%) did not reach statistical significance (P = 0.063) (Figure 6).

### 3.5 Model performance by Ophthalmic Subspecialty

Analysis of model accuracy across ten subspecialties revealed distinct performance profiles. Gemini-3-Flash demonstrated robust performance, achieving the highest accuracy in domains such as Glaucoma (93.2%), Cornea/ED (86.1%), and Pediatric Ophthalmology/Strabismus (70.4%). In contrast, GPT-5 showed notably lower accuracy in specific areas, particularly in Trauma (61.9%) and Pediatric Ophthalmology/Strabismus (48.3%). DeepSeek-R1 performed strongly in Trauma (86.9%) and Uveitis (86.1%).

**FIGURE 7:**
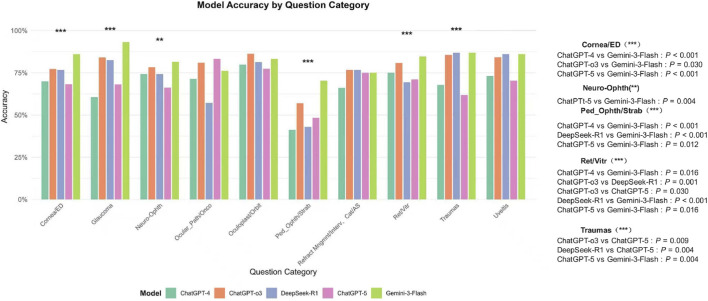
Model Accuracy by Ophthalmic Subspecialty. This chart compares the diagnostic accuracy of five large language models—GPT-4, GPT-o3, DeepSeek-R1, GPT-5, and Gemini-3-Flash—across ten specialized domains. Gemini-3-Flash demonstrated robust performance, leading in Glaucoma (93.2%), Cornea/ED (86.1%), and Pediatric Ophthalmology/Strabismus (70.4%), while DeepSeek-R1 performed strongly in Traumas (86.9%) and Uveitis (86.1%). Statistical analysis revealed that in the Traumas and Uveitis subspecialties, both DeepSeek-R1 and Gemini-3-Flash significantly outperformed GPT-5 (P < 0.01 and P < 0.05, respectively). In Retina/Vitreous (Ret/Vitr), Gemini-3-Flash maintained a significant advantage over GPT-4, DeepSeek-R1, and GPT-5 (all P < 0.01). Conversely, GPT-5 showed notably lower accuracy in Traumas (61.9%) and Pediatric Ophthalmology (48.3%). No statistically significant differences were observed in Ocular Pathology/Oncology or Refractive/Cataract surgery categories, and notably, no significant performance gap was found between GPT-4 and GPT-5 across any subspecialty. Significance levels are indicated by asterisks (**P < 0.01; ***P < 0.001), with detailed pairwise P-values listed in the right-hand panel.

In the Trauma subspecialty, both DeepSeek-R1 and Gemini-3-Flash significantly outperformed GPT-5 (p < 0.01), whereas no significant difference was observed between GPT-4 and GPT-5. In Uveitis, DeepSeek-R1 and Gemini-3-Flash also demonstrated significantly higher accuracy than GPT-5 (p < 0.05). In Retina/Vitreous, Gemini-3-Flash maintained a significant advantage over GPT-4, DeepSeek-R1, and GPT-5 (all p < 0.01). Comparisons in Ocular Pathology/Oncology and Refractive/Cataract surgery did not reveal statistically significant differences. Notably, no statistically significant difference was found between GPT-4 and GPT-5 across any subspecialty (Figure 7).

## 4 Discussion

This study presents the first systematic evaluation of five cutting-edge LLMs—GPT-4, GPT-5, GPT-o3, Gemini-3-Flash, and DeepSeek-R1—in ophthalmology-specific question-answering, with a particular focus on output stability. We assessed the consistency (i.e., within-model agreement) of model responses across repeated tests, a fundamental prerequisite for reliable performance assessment. Our results reveal several key findings.

### 4.1 The stability (i.e., internal consistency) of model decisions serves as a fundamental prerequisite for reliable performance assessment

This study focused on the consistency (within-model agreement) of model responses across repeated tests, a critical prerequisite for assessing the reliability of model performance. In medical AI evaluation, reproducibility and stability are essential to ensure that reported performance reflects underlying model competence rather than stochastic variation, particularly in high-stakes clinical domains (Topol, 2019). Our results demonstrate that GPT-o3 exhibits the highest decision stability (κ = 0.966), outperforming DeepSeek-R1 (κ = 0.904), Gemini-3-Flash (κ = 0.860), and GPT-4 (κ = 0.842). In contrast, GPT-5 demonstrated substantially lower internal consistency (κ = 0.668), reflecting greater variability in its repeated responses under identical conditions.

Across all models, a decline in consistency was observed at the highest difficulty level (Level 4), suggesting that the increased complexity of the task places greater demands on the model’s decision-making processes, which in turn exacerbates variability in the output. This decline was most pronounced for GPT-4 and GPT-5, whereas GPT-o3 maintained relatively high agreement even under the most challenging conditions. Such findings align with prior observations that increasing clinical complexity can exacerbate instability in LLM output (Singha et al., 2023; Wang et al., 2024).

Previous studies have reported limited output stability in GPT-4 during ophthalmology question-answering tasks, raising concerns regarding the reproducibility of its responses across repeated assessments. By employing Fleiss’ kappa as a formal measure of agreement, the present study not only corroborated these findings but also extended the analysis to a broader suite of contemporary models. To our knowledge, this work provides the first quantitative evidence of response reproducibility across current state-of-the-art models. Our results demonstrated that GPT-o3 significantly enhanced decision stability compared to its predecessors.

Subspecialty-level analyses further revealed that internal consistency varies substantially across clinical domains, indicating that decision stability is influenced not only by model architecture but also by the cognitive structure of the task itself. Subspecialties with relatively standardized diagnostic pathways (e.g., Glaucoma and Retina) exhibited consistently high agreement across models, often approaching perfect reproducibility. By contrast, those characterized by greater diagnostic heterogeneity or interpretative ambiguity (e.g., Pediatric Ophthalmology/Strabismus and Ocular Pathology/Oncology) showed greater variability in kappa scores. This domain-dependent variability echoes findings from prior benchmarking studies in ophthalmology and radiology, in which performance consistency differed markedly across subspecialties (Demir, 2024; Nguyen et al., 2025).

Notably, GPT-5 exhibited substantial dispersion in subspecialty-level agreement, yielding low-to-moderate Kappa values across several prompt configurations. A negative Kappa value was calculated for GPT-5 in the Ocular Pathology/Oncology domain under Prompt 1. Although GPT-o3, which was the most consistent model under Prompt 0, also exhibited a decline in performance within this subspecialty, the limited sample size (n = 7) may have influenced the results for both models. Nevertheless, these findings suggest that GPT-5 is more susceptible to contextual framing and task-specific uncertainties. The near-perfect consistency maintained by other models in this same subspecialty indicates that the task itself is not inherently ambiguous, but rather highlights disparities in the robustness of the models’ underlying decision-making processes. Having established baseline output stability, we next examined factual accuracy.

### 4.2 A clear performance stratification exists among the models

Building upon the confirmed stability of the models, we further analyzed their response accuracy. Significant differences in accuracy were observed among the models (p < 0.001). Gemini-3-Flash demonstrated the highest overall performance (83.3%), followed by GPT-o3 (79.2%) and DeepSeek-R1 (74.4%). GPT-4 (69.9%) and GPT-5 (69.1%) achieved the lowest accuracies, with no significant difference between them. These results suggest that newer, reasoning-optimized models (Gemini-3-Flash, GPT-o3, and DeepSeek-R1) consistently outperform both GPT-4 and GPT-5, extending previous findings. Early reports indicated that DeepSeek models achieved accuracy comparable to GPT-4 in specialized areas such as neuro-ophthalmology (Li X. et al., 2025). Our study provides preliminary evidence that this performance advantage holds across a broader range of ophthalmic knowledge. Historically, the transition from GPT-3.5 to GPT-4 marked a significant leap in medical AI, with accuracy gains of 15%–20% across various ophthalmic benchmarks (Taloni et al., 2023; Wei et al., 2025; Haddad and Saade, 2024). However, our results reveal a counter-trend: the substantial performance gains anticipated with GPT-5 were not realized in this specialized domain. Despite its positioning as a major advancement in healthcare AI—with claims of enhanced medical reasoning and clinical decision support (GPT, 2025; GPT-5, 2025)—GPT-5’s accuracy (69.1%) failed to surpass GPT-4. This aligns with recent findings in gastrointestinal oncology, where GPT-5 did not outperform GPT-4o in tumor board decision consistency (Dinc et al., 2025).

The observed performance patterns across the five models reveal a nuanced landscape of LLM capabilities in ophthalmology. In Oculoplastics and Orbital Surgery, GPT-o3 achieved the highest accuracy (86.3%), outperforming Gemini-3-Flash (83.3%) and DeepSeek-R1 (81.4%), thereby establishing itself as a leading model in this anatomical domain. Similarly, in Neuro-ophthalmology—requiring complex localization and reasoning—Gemini-3-Flash maintained the highest accuracy (81.5%), followed by GPT-o3 (78.4%), while GPT-5 trailed significantly (66.2%).

These findings demonstrate a notable advancement over earlier iterations of the Gemini series. Previous research on Gemini in ophthalmology remains limited (Masalkhi et al., 2024; Mihalache et al., 2024; Carlà et al., 2024). Specifically, Botross et al. reported that an earlier version achieved an overall accuracy of 62.4% across 250 ophthalmic questions, with particularly poor performance in retina-related topics (24.0%) (Botross et al., 2024). In contrast, our study shows that Gemini-3-Flash has largely overcome these limitations, securing a leading position in Retina/Vitreous (84.7%) and other areas.

An analysis of performance consistency further distinguishes the top-tier models. While DeepSeek-R1 matched or exceeded the best models in Trauma and Uveitis, it exhibited notable volatility, dropping to 57.1% in Ocular Pathology/Oncology—the lowest performance recorded in that category. In contrast, GPT-o3 demonstrated remarkable stability, consistently placing second across most subspecialties, including Cornea, Glaucoma, and Retina/Vitreous, while ranking highest primarily in Oculoplastics. These findings suggest that although Gemini-3-Flash achieves peak performance, GPT-o3 offers a highly reliable alternative with lower variance across ophthalmic domains.

However, these findings warrant cautious interpretation. The uneven distribution of questions across subspecialties may partly account for the observed advantages among the models, which may also reflect their evolving architectural strategies, including the reinforcement learning-based reasoning frameworks employed in GPT-o3 and DeepSeek-R1, as well as the optimized contextual processing in Gemini-3-Flash (OpenAI, 2025b; Open AI, 2025a; Gemini 3, 2025a).

### 4.3 Limited effect of prompting strategies

We examined whether alternative prompting approaches affected model performance. Across the five models, there were no material differences in accuracy, whether questions were posed as a simple direct prompt (Prompt 0) or with added role/background priming (Prompt 1). For knowledge-oriented choice items, once the prompt explicitly required a definitive answer, appending role specifications or step-by-step instructions did not further improve accuracy. In other words, performance on these tasks appears to depend primarily on the models’ underlying knowledge and reasoning capacity rather than on minor variations in phrasing. This pattern is consistent with the observations of Peng et al. (2025), who reported that some LLMs have internalized CoT-like response patterns, thereby reducing, to some extent, reliance on explicit prompt engineering (Peng et al., 2025). Practically, clinicians employing LLMs for exam-style question answering need not invest in elaborate prompt designs; clear, direct queries are typically sufficient.

### 4.4 Divergent performance across task difficulty

The findings of this study reveal a critical trend: increasing task complexity does not uniformly degrade the performance of ever LLM. The observed statistical stability in models like Gemini-3-Flash and GPT-5 (p > 0.05) suggests that next-generation architectures may possess greater robustness against such complexity. Notably, Gemini-3-Flash maintained high accuracy without significant fluctuations as difficulty increased. This consistency could stem from optimized adaptive inference allocation and advanced instruction-tuning, which likely enable the model to preserve logical coherence across diverse medical contexts (Gemini 3, 2025b). In contrast, the performance variability observed in earlier models (e.g., GPT-4) indicates that traditional architectures may be more susceptible to interference from extraneous information present in complex clinical queries (GPT-4, 2025).

Notably, both GPT-o3 and DeepSeek-R1 exhibited a non-monotonic performance pattern, peaking at Level 3 while delivering suboptimal results at Level 2. This phenomenon can be partially explained through the lens of CoT reasoning costs. For intuitive clinical questions with shorter logical pathways, the complex internal reasoning chains may introduce extraneous logical “noise,” potentially leading to systematic overthinking (Guo et al., 2025). However, as task difficulty escalates to Level 3, the inherent advantage of their “think-before-answer” architecture becomes more prominent. The systematic step-by-step decomposition likely helps these models avoid common logical pitfalls in medical diagnosis and treatment (OpenAI, 2025). DeepSeek-R1’s effective handling of complex medical tasks, compared to its performance on simpler ones, not only points to the promise of open-source models in this domain but also emphasizes their rapid development and suitability for targeted fine-tuning and knowledge integratio (Haddad and Saade, 2024; Mikhail et al., 2025).

### 4.5 Limitations

This study has several limitations. First, the sample was restricted to 300 single-best-answer multiple-choice questions from a single question bank, which may limit the generalizability of our findings. Furthermore, as this study utilized a random sampling method, the distribution of questions across subspecialties is uneven; for instance, the Ocular Pathology/Oncology domain contains only seven items. This imbalance may introduce bias into the results, which should therefore be carefully considered when interpreting the findings. Second, our evaluation focused exclusively on a closed-ended question–answering paradigm. We did not examine open-ended problem solving, clinical case analysis, or multimodal scenarios (e.g., integration of imaging data). As a result, the purely text-based evaluation setting simplifies the complexity of real-world clinical decision-making, which typically requires the synthesis of multiple information sources, thereby constraining the direct clinical applicability of our results. Finally, external evidence suggests that LLM performance can vary depending on factors such as Application Programming Interface (API) platform, deployment configuration, and time of access. Accordingly, our findings should be interpreted as reflecting performance under the specific conditions evaluated in this study.

### 4.6 Conclusions and outlook

This study presents the first systematic evaluation of five cutting-edge LLMs—GPT-4, GPT-5, GPT-o3, Gemini-3-Flash, and DeepSeek-R1—in the domain of ophthalmology question-answering, with a specific focus on output stability and factual accuracy. The findings provide several key insights into the current state of medical AI.

First, GPT-o3 demonstrated the best overall performance balance, achieving high accuracy (79.2%) and the highest response stability (κ = 0.97). Its advantage aligns with an internal ‘think-before-respond’ reasoning architecture, indicating that iterative model development may contribute to the advancement of medical AI. Second, Gemini-3-Flash achieved the highest overall accuracy (83.3%) and demonstrated robust performance across all difficulty levels and various subspecialties, such as Glaucoma and Retina. Third, the open-source DeepSeek-R1 showed considerable potential, exceeding GPT-4 on moderately difficult tasks, making it a promising alternative for resource-constrained settings. In contrast, GPT-5 not only failed to surpass GPT-4 in accuracy but also exhibited substantially lower internal consistency. Furthermore, prompt strategies had no statistically significant impact on performance, indicating that these models rely more on underlying knowledge than on specific phrasing.

Based on these findings, we propose that high-stakes clinical decisions must retain human oversight. AI-assisted tools should prioritize models with stable and accurate performance, such as GPT-o3 and Gemini-3-Flash. However, it is important to note that this study merely assesses the potential of these models in structured ophthalmology question-answering tasks. A genuine clinical decision support system must undergo far more rigorous evaluation, including but not limited to: robustness against hallucinations, the ability to handle incomplete or ambiguous inputs, integration of multimodal data, interpretability of decisions, and prudent behavior in safety-critical scenarios. The findings of this study should not be interpreted as indicating that these models are qualified for clinical deployment. Instead, they provide a preliminary performance baseline for the future development of more reliable and specialized AI-assisted tools in ophthalmology. Future work will focus on developing multimodal models that integrate medical imaging with text and rigorously evaluating their reliability, safety, and ethical compliance in real-world clinical environments. While certain models currently lead our evaluation, future efforts should balance performance, computational efficiency, and cost-effectiveness to identify the most suitable solutions for clinical implementation. In summary, while the results highlight the capabilities of specific architectures, further research into multimodal integration and real-world clinical application is necessary to determine the practical utility of these tools.

## Data Availability

The original contributions presented in the study are included in the article/supplementary material, further inquiries can be directed to the corresponding authors.
